# Chiropractic and self-care for back-related leg pain: design of a randomized clinical trial

**DOI:** 10.1186/2045-709X-19-8

**Published:** 2011-03-22

**Authors:** Craig A Schulz, Maria A Hondras, Roni L Evans, Maruti R Gudavalli, Cynthia R Long, Edward F Owens, David G Wilder, Gert Bronfort

**Affiliations:** 1 Northwestern Health Sciences University, Wolfe-Harris Center for Clinical Studies, 2501 West 84th Street, Bloomington, MN 55431, USA; 2 Palmer College of Chiropractic, Palmer Center for Chiropractic Research, 741 Brady Street, Davenport, IA 52803-5209, USA; 3 University of Iowa, Biomedical Engineering Department, 1402 Seamens Center for Engineering Arts and Sciences, Iowa city, Iowa 52242-1527, USA

## Abstract

**Background:**

Back-related leg pain (BRLP) is a common variation of low back pain (LBP), with lifetime prevalence estimates as high as 40%. Often disabling, BRLP accounts for greater work loss, recurrences, and higher costs than uncomplicated LBP and more often leads to surgery with a lifetime incidence of 10% for those with severe BRLP, compared to 1-2% for those with LBP.

In the US, half of those with back-related conditions seek CAM treatments, the most common of which is chiropractic care. While there is preliminary evidence suggesting chiropractic spinal manipulative therapy is beneficial for patients with BRLP, there is insufficient evidence currently available to assess the effectiveness of this care.

**Methods/Design:**

This study is a two-site, prospective, parallel group, observer-blinded randomized clinical trial (RCT). A total of 192 study patients will be recruited from the Twin Cities, MN (n = 122) and Quad Cities area in Iowa and Illinois (n = 70) to the research clinics at WHCCS and PCCR, respectively.

It compares two interventions: chiropractic spinal manipulative therapy (SMT) plus home exercise program (HEP) to HEP alone (minimal intervention comparison) for patients with subacute or chronic back-related leg pain.

**Discussion:**

Back-related leg pain (BRLP) is a costly and often disabling variation of the ubiquitous back pain conditions. As health care costs continue to climb, the search for effective treatments with few side-effects is critical. While SMT is the most commonly sought CAM treatment for LBP sufferers, there is only a small, albeit promising, body of research to support its use for patients with BRLP.

This study seeks to fill a critical gap in the LBP literature by performing the first full scale RCT assessing chiropractic SMT for patients with sub-acute or chronic BRLP using important **patient-oriented **and **objective biomechanical **outcome measures.

**Trial Registration:**

ClinicalTrials.gov NCT00494065

## Background

Low back pain (LBP) is well recognized as a significant individual and societal burden with lifetime prevalence estimates of up to 80%,[1,2] contributing to rising health care costs in the United States that are now estimated to exceed $100 billion annually[3]. Back-related leg pain (BRLP) is a common variation of LBP,[4-6] with lifetime prevalence estimates as high as 40%[5]. A population-based survey from the Netherlands reported a one year prevalence of 13-24% for radiating leg symptoms[6]. Often disabling, BRLP accounts for greater work loss, recurrences, and costs than uncomplicated LBP[7-10]. Further, the lifetime incidence of surgery is 10% for those with severe BRLP, compared to 1-2% for those with LBP[10]. By far the most common reason for back surgery is herniated lumbar disc, a common cause of BRLP[11].

In the US, half of those with back-related conditions seek complementary and alternative medicine (CAM) treatments, the most common of which is chiropractic care[12,13]. While there is preliminary evidence suggesting chiropractic spinal manipulative therapy is beneficial for patients with BRLP, there is insufficient evidence currently available to assess the effectiveness of this care[14,15].

### Definition of Back-Related Leg Pain (BRLP)

BRLP is defined as the constellation of symptoms characterized by unilateral or bilateral radiating pain originating in the lumbar region and traveling into the proximal or distal lower extremity with or without neurological signs[16,17]. BRLP includes both radicular and nonradicular radiating pain. Radicular radiating pain is defined as pain caused by a lumbar nerve root lesion, resulting in pain radiating from the back into the dermatome of that root along the femoral or sciatic nerve distribution. Nonradicular radiating pain is defined as pain radiating from the back into the leg in a nondermatomal pattern[18].

### Etiology of Back-Related Leg Pain

BRLP of radicular origin caused by lumbar nerve root irritation can be secondary to a variety of causes including one or more herniated lumbar discs[19]. Disc herniation can lead to compression or traction of a nerve root and subsequent intra-neural inflammation[20,21]. Inflammation may also be caused by biochemical mechanisms. For example, the breakdown products from a degenerating nucleus pulposis may leak into the epidural space and result in "chemical radiculitis" of the nerve root[22]. Other possible causes of lumbar nerve root irritation are spinal stenosis, nerve root canal narrowing, and synovial cysts[23]. BRLP of nonradicular origin is caused by biomechanical dysfunction or pathological changes in the paraspinal muscles, ligaments, discs, facet joints, or other structures of the lumbar motion segments[24].

### Interventions

Conservative or non-operative management is the first line of therapy for most BRLP patients[25]. Surgery is a more costly treatment strategy and is only indicated in patients with progressive neurological deficits or unmanageable pain[26]. Some of the most commonly used conservative approaches are physical treatments such as chiropractic spinal manipulative therapy[26].

#### Chiropractic Spinal Manipulative Therapy (SMT)

The most common reason patients pursue CAM treatments in the US is for back pain conditions[13]. An estimated 20-30% of these patients seek care from chiropractors,[12,27] making it the most frequently sought CAM treatment for back disorders[12,13]. SMT is the most frequently used treatment modality in chiropractic practice,[28] and chiropractors are the primary providers of SMT in North America[29].

Several systematic reviews have evaluated SMT for LBP conditions[19,30-32] and are in general agreement that SMT is one of several treatment options of modest effectiveness for LBP. Two earlier literature reviews focused *specifically *on BRLP, or sciatica[14,15].

A randomized clinical trial by Santilli et al (n = 102) assessed chiropractic SMT versus sham manipulation for patients with acute sciatica and confirmed disc herniation[33]. Significant differences were observed between groups in both back and leg pain in favor of the active SMT group at the 6 month follow-up period. The percentage of cases becoming pain-free was 28% vs. 6% for local pain (p < .005) and 55% vs. 20% for radiating pain (p < .0001). Importantly, no adverse events were observed. This study offers the most compelling evidence to date regarding the efficacy of chiropractic SMT for BRLP, specifically acute cases. The evidence is not clear, however, regarding efficacy for patients with sub-acute and chronic BRLP.

In 2004, Haas et al reported a prospective observational cohort study (n = 2870) of **chronic **LBP, which included patients with radiating pain below the knee. They found the subgroup of patients with radiating pain to experience better long term outcomes with chiropractic care (including SMT) than medical care[34]. These results are supported by subgroup analyses of two trials of SMT for chronic LBP (including BRLP) performed by the investigators of this trial[35,36]. Both trials observed medium to large effect sizes for pain reduction in favor of SMT in the patients with BRLP. While there is preliminary evidence suggesting chiropractic SMT is beneficial for patients with BRLP, there is insufficient evidence currently available to assess the effectiveness of this care[14,15,37]. Our study addresses this need.

#### Home Exercise Program (HEP) (Minimal Intervention Comparison)

Given the lack of research investigating conservative treatments for BRLP, there are many questions worth investigating. In the absence of an established, standard treatment for BRLP, it is important to compare the magnitude of SMT treatment effects to those of no treatment, waiting list, or minimal intervention comparison group. For this study, we have chosen the latter comparison.

Patient education has been used successfully in several studies as a minimal intervention comparison group, including several by investigators conducting this study[38-42]. Defined as any set of planned educational activities designed to improve patient's health behaviors and/or health status,[43] patient education has become an important and recommended intervention in clinical practice[44-48]. A systematic review by Enger et al[43] found strong evidence for an educational intervention compared to no intervention in acute and sub-acute LBP patients; however, due to a lack of research, its utility for chronic LBP conditions remains unclear.

Our group conducted two pilot studies to develop study protocols and assess recruitment feasibility [42,49] and found recruitment of BRLP patients to be challenging. For this reason, we chose to employ a two site approach to facilitate recruitment.

The objective of this study is to evaluate the relative effectiveness of **chiropractic spinal manipulative therapy (SMT) plus a home exercise program (HEP) **to a **HEP alone (minimal intervention comparison)**. Using two sites enhances the pool of potential participants and has the added benefit to increase generalizability.

This article describes the study protocol for the clinical trial currently in progress.

## Methods/Design

### Study Overview

**Figure 1:**
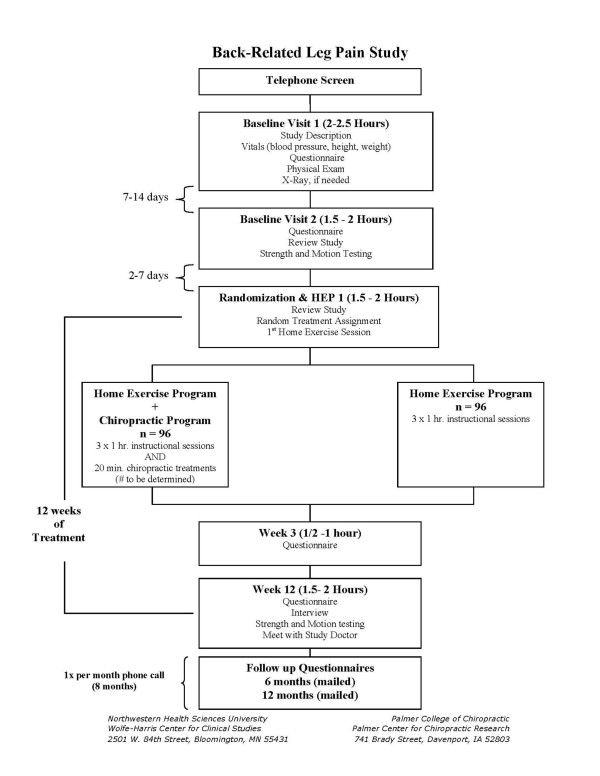
**Participant Flow chart**. Participant flow, study visits, and evaluations.

This study is a two-site, prospective, parallel group, observer-blinded randomized clinical trial (RCT). It compares two interventions: chiropractic spinal manipulative therapy (SMT) plus a home exercise program (HEP) to a HEP alone (minimal intervention comparison) for patients with subacute or chronic back-related leg pain. Participant flow is illustrated in Figure 1. Data collection measures and study protocols are standardized across sites. The Office of Data Management and Biostatistics at the Palmer Center for Chiropractic Research (PCCR) serves as the Data Coordinating Center (DCC) with a web-based interface for centralized data handling and treatment assignment. This ongoing study began participant recruitment at the Wolfe-Harris Center for Clinical Studies (WHCCS) and PCCR in May 2007. Institutional review boards of all participating institutions have approved the research and informed consent is obtained from all participants.

### Study Population

A total of 192 study patients will be recruited from the Twin Cities, MN (n = 122) and Quad Cities area in Iowa and Illinois (n = 70) to the research clinics at WHCCS and PCCR, respectively. Specific subgroups of LBP (i.e., BRLP) patients can be difficult to recruit[49]. Multiple recruitment strategies are used based on investigators experience[50] and pilot studies for patients with BRLP[42,49]. The multi-method recruitment strategy includes: mass media, mass mailings, and clinical referrals.

### Inclusion/Exclusion Criteria

Inclusion and exclusion criteria are presented in Table 1. Participant flow data is recorded in accordance with the Consolidated Standards of Reporting Trials (CONSORT) statement[51] and will be reported with final trial results.

**Table 1:** Inclusion/Exclusion Criteria

Inclusion Criteria	Exclusion Criteria
**Back-related leg pain > 3 (0-10 scale)**	**Ongoing treatment for leg or low back pain by other health care providers**
**Sub-acute or chronic back-related leg pain **defined as **current episode > 4 weeks duration**	**Progressive neurolo gical deficits or cauda equine syndrome**
**Back-related leg pain classified as 2, 3, 4, or 6 using the Quebec Task Force (QTF) Classification system**[16]. This includes radiating pain into the proximal or distal part of the lower extremity, with or without neurological signs, with possible compression of a nerve root.	**QTF 1 **(pain without radiation), **5 **(spinal fracture), **and 11 **(other diagnoses including visceral diseases, compression fractures, metastases). These are serious conditions not amenable to the conservative treatments proposed[16,25,110].
**21 years of age and older**	**QTF 7 **(spinal stenosis syndrome characterized by pain and/or paresthesias in one or both legs aggravated by walking)[16].
**Stable prescription medication plan **(No changes in prescription medications that affect musculoskeletal pain in the previous month.)	**Uncontrolled hypertension or metabolic disease**
	**Blood clotting disorders**
	**Severe osteoporosis**
	**Inflammatory or destructive tissue changes of the spine**
	**QTF 8 and 9 **(surgical lumbar spine fusion) or patients with multiple incidents of lumbar surgery. This is a subgroup of low back pain patients which generally have a poorer prognosis[111].
	**QTF 10 **(chronic pain syndrome)
	**Pregnant or nursing women**
	**Current or pending litigation**. Patients seeking financial compensation tend to respond differently to treatment[112].
	**Inability to read or verbally comprehend English**
	**Evidence of narcotic or other drug abuse**
	**Unwillingness to postpone all other types of manual therapy treatment for LBP or BRLP except those provided in the study for the duration of the study period**.

### Eligibility Determination

#### Phone Screen

Potential participants respond to recruitment materials by contacting the research centers and are screened for initial eligibility criteria by trained interviewers using a computer-assisted telephone interview module in the database system. Participants meeting the eligibility criteria are scheduled for an in-person screening interview and physical examination.

#### Baseline Evaluation One (BEV1)

Individuals who qualify for baseline evaluation attend the first of three baseline appointments which includes informed consent and HIPAA processes. Patients complete a self-report questionnaire (described below under outcome measures), health history, and physical examination (neurological examination, orthopedic tests, inspection and palpation of the thoracic and lumbar spine and lower extremities). Plain film radiographs, bone mineral density scans, and previous medical records are obtained as needed. Participants who qualify and agree to participate are scheduled for a second baseline evaluation to occur within 7-14 days. Chiropractic and allopathic practitioners participate in patient examinations and weekly case reviews to determine participant eligibility. Medical clinicians also provide clinical consultation and "rescue medication" as needed for patients with acute exacerbations.

#### Case Review

Prior to the second baseline evaluation, investigators and study clinicians review each case at weekly case review meetings for clinical eligibility determination. A web-based form, designed to prevent errors in eligibility determination, is completed for each patient. The web form confirms patient eligibility by cross-referencing inclusion and exclusion criteria with clinical and patient reported measures collected at the BEV1. The review committee reaches consensus on every case and either recommends exclusion, inclusion (i.e., continuation of baseline evaluation) or follow-up for further tests.

#### Baseline Evaluation Two (BEV2)

The second baseline evaluation includes a review of informed consent and a self-report questionnaire. Examiners perform a suite of objective biomechanical assessments, which take about 1 hour to complete. The testing methods are described in detail below. Examiners are trained and certified annually by investigators using video recording of testing procedures to review and document competency in patient instruction, equipment operation, and protocol adherence. Participants then schedule for the third baseline appointment.

#### Randomization and first treatment

This visit occurs 2-7 days following BEV2, and is intended to provide additional time for potential participants to consider participation and also allows time for individuals to assess their tolerance for the biomechanical outcome measures. At this appointment, participants are randomly allocated to intervention and go on to their first treatment.

### Randomization

An adaptive computer-generated randomization scheme is used to minimize group differences in 7 baseline factors over all patients enrolled at both sites[52]. The scheme attempts to provide the best balance of the following baseline characteristics: age (< 50 years vs. ≥ 50 years); duration of leg pain at the BEV2 (< 12 weeks, 12-25 weeks, 26-51 weeks, 1-5 years, and > 5 years); presence or absence of neurological signs in the leg (QTF classification 2 or 3 vs. classifications 4 or 6); distress at the BEV2 (SF-36 items positive vs. negative for distress); positive straight leg raise test at BEV2 (≤45 degrees vs. ≥45 degrees); amount of time spent driving a vehicle (< 2 hours several times per week vs. 2 or more hours several times per week); and leg pain aggravation with coughing or sneezing (no vs. yes). All study personnel are blinded to upcoming treatment assignments and the biomechanics objective examiners are blinded to treatment assignment throughout the course of the trial. The algorithm was programmed by the DCC Database Programmer and the database is maintained by the DCC Data Manager. The back-up treatment assignment protocol is by predetermined sequentially numbered, opaque envelopes prepared by the DCC Data Manager and maintained by the Project Manager at each site.

### Treatment

Treatment protocols for this study were developed and refined in our pilot studies[42,49]. Standardized forms are used to document treatment procedures and reviewed to monitor protocol deviations. The time frame for treatment is 12 weeks; this is based on results of previous[36,42,49,53,54] and ongoing studies and consensus of participating clinicians. All treatments are provided in the research clinics of WHCCS and PCCR.

#### Chiropractic Spinal Manipulative Therapy (SMT)

To provide for study results that might be more generalizable to the private practice setting, we decided to allow the treating chiropractor to determine the number and frequency of treatments, based on patient-rated symptoms, disability, palpation, and pain provocation tests[55]. Up to 20 treatments may be provided over the 12 week treatment period, with each treatment visit lasting from 10-20 minutes. In our pilot study, the mean number of treatments provided was 15[42].

Chiropractic assessment and treatment follow standardized protocols. The spine and pelvis are evaluated by the individual chiropractor using static and motion palpation and pain provocation tests shown to have acceptable reliability[55]. Treatment includes manual spinal manipulation and mobilization. Light soft tissue techniques (i.e., active and passive muscle stretching and ischemic compression of tender points) and hot and cold packs are used as indicated to facilitate the manual therapy. For spinal manipulation, the chiropractor's contact hand is placed over an osseous process, muscle, or ligament and the vertebral or sacroiliac joint of interest is taken to the end of its physiological range of motion. The chiropractor then applies a high velocity, low amplitude thrust (HVLA) to the joint. Patients with severe pain or leg pain of radicular origin may not tolerate the dynamic nature of HVLA manipulation. These patients are treated with low velocity mobilization techniques described in our previous work (i.e., low velocity joint mobilization, flexion-distraction, and neuromuscular techniques)[35]. Similar protocols for delivering chiropractic manipulation and mobilization[36,41,42,42,49,56,57] have been used in previous and ongoing RCTs by the investigators. Patient and provider adherence rates have varied from 91-97%, indicating the protocols are acceptable to both patients and providers.

#### Home Exercise Program

Patient education is provided by trained therapists under the supervision of licensed chiropractic clinicians. Patients attend four, 1-hour, one-on-one sessions. Previous research suggests that at least 2.5 hours is necessary for individual patient education to be effective[43]. The goals of the program include improving patients' understanding of their back problems, reducing unwarranted concerns about serious outcomes, empowering patients to take actions expediting return to normal activities (through self-care postures and exercise), reducing the risk of subsequent back problems, and minimizing dependency on health care providers[44,58].

The sessions follow a standardized approach but are individualized to meet the patient's needs specific to their lifestyle, fitness level, and clinical characteristics. Patients are taught methods for developing spinal posture awareness for their individual activities of daily living, such as lifting, pushing and pulling, sitting, and getting out of bed[59,60]. Based on their abilities and clinical evaluation, patients are also shown exercises to enhance mobility and increase trunk endurance. These may include flexion/extension motion cycles, hip/knee stretches, prone press-ups (back extension), slow lunges, abdominal curl-ups, side bridge variations, and leg and arm extension variations[61]. They are encouraged to do the exercises at home daily.

Patients are given simple instructions for the exercises. At visits 2, 3, and 4, therapists review the exercises with patients to ensure proper form. An adaptation of the *Back in Action *book[58] is given to all patients, emphasizing the "biopsychosocial message," which encourages movement and restoration of normal function and fitness[40,43].

### Rescue Medication and Surgical Consultation

Prescription strength rescue medication is available for patients experiencing severe pain and is prescribed as needed by a medical doctor. A clinical decision-making rule agreed upon by the medical clinicians is used to manage acute exacerbations. Patients may receive NSAIDs, opioids, and/or muscle relaxants. Any patient who demonstrates progressive neurological signs or severe, intractable pain is removed from study treatment and referred for surgical consultation. These patients continue to be followed and remain in the intention-to-treat analysis.

### Data Collection

Self-reported outcome measures are collected at baseline and 3, 12, 26, and 52 weeks post-randomization; blinded objective biomechanical outcome measures are assessed by blinded examiners at baseline and 12 weeks. Qualitative patient interviews are conducted at 12 weeks.

#### Week 3 and 12 Evaluations

Three and 12 weeks after randomization, participants complete self-report questionnaires assessing primary and secondary outcome measures. At the week 12 evaluation, trained research assistants blinded to patients' treatment assignment perform the same objective assessments performed at BEV2. Qualitative interviews are also conducted at week 12. Follow-up rates at similar time points in previously published studies by the investigators have been 90-97%[41,42,49,53] and 91-100% in ongoing randomized trials.

#### Week 26 and 52 Evaluations

At 26 and 52 weeks post-randomization, participants are mailed self-report questionnaires measuring primary and secondary outcomes. Self-addressed, postage paid envelopes are provided to return completed questionnaires. Data collection rates for similar time points vary from 85-100% in previous and ongoing studies by the investigators[42,53].

#### Participant Flow Data

Patient flow characteristics (i.e., number evaluated, disqualified, etc.) are monitored and reported according to the CONSORT guidelines[62].

#### Demographic and Clinical Information

Important demographic and clinical information is collected for every participant through baseline self-report questionnaires, interviews, and physical examinations.

### Outcome Measures

Outcome measures are collected both by patient self-report and blinded objective assessment, and are consistent with suggestions made for the standardized measurement of outcomes in LBP clinical trials[63]. The patient burden is 30-40 minutes for BEV1 questionnaires, and 15-25 minutes for subsequent questionnaires. Blinded objective biomechanical measurements take approximately 1 hour to collect.

#### Patient Self-Report Outcome Measures

##### Primary Outcome Measure

##### Leg Pain

Previous research conducted by the investigators found that pain is one of the most important outcome measures for patients with BRLP[64]. Patients are asked to rate their typical level of leg pain during the past week on an ordinal 11-box scale (0 = no pain, 10 = the worst pain possible)[65]. Several studies have shown that ordinal pain scale measures perform as well as the 10 cm Visual Analog Scale (VAS),[65] a simple, frequently used valid assessment of variation in pain intensity[66,67] and a reliable measure of treatment efficacy[68]. The advantage of the 11-box scale over the VAS is that it is easier to administer and score[66].

##### Secondary Outcome Measures

##### Low Back Pain

Patients with BRLP typically experience low back symptoms[42,49]. Patients are asked to rate their typical level of LBP during the past week on an ordinal 11-box scale as described above.

### Bothersomeness of Symptoms

Using a 0 to 5 scale (0 = not at all bothersome, 5 = extremely bothersome), five items are measured: 1) back pain, 2) buttock pain 3) leg pain, 4) numbness or tingling in leg(s), and/or feet, 5) weakness in leg(s) and/or feet (such as difficulty lifting foot). A BRLP bothersomeness index is calculated by summing the five symptom ratings in a scale (0-25). This index has good internal consistency, construct validity, and responsiveness in BRLP patients[69].

### Frequency of Symptoms

Frequency of the same symptoms described for bothersomeness is measured on a 0 to 5 scale (0 = none of the time, 5 = all of the time). By summing the five symptom ratings, a frequency index is constructed resulting in a 0 to 25 scale. This index has been shown to have good internal consistency, construct validity, and responsiveness[63,69].

### Disability

Disability is measured with the Modified Roland Morris Scale, a 23-item questionnaire that measures the degree to which BRLP restricts patients' daily activities[70,71]. It has a high level of internal consistency, construct validity, and responsiveness, and is scored by simply summing the number of "yes" and "no" answers[69]. A percentage score is calculated based on the number of "yes" scores.

### General Health Status

Functional health status is measured by the reliable, valid, and widely used Medical Outcomes Study Short Form 36-item Health Survey (SF-36v2), which measures eight domains: physical functioning, social functioning, mental health, energy and vitality, pain, general health, and role limitations due to physical and emotional problems[72-74]. This index has been shown to have good internal consistency, construct validity, and responsiveness in sciatica patients[69].

### Fear Avoidance Beliefs

The Fear Avoidance Beliefs Questionnaire (FABQ) was developed to study the relationship between LBP, fear avoidance beliefs and behaviors, and chronic disability[75]. This self-report instrument consists of 16 items, each item answered on a 7 point Likert agreement scale that yields two subscales: work and physical activity. High levels of test-retest reliability have been reported for the work subscale (ICC = .90) and physical activity subscale (ICC = .77)[75,76].

### Patient Satisfaction

Patient satisfaction is measured on 7-point scale (1 = poor, 7 = excellent) using eight questions addressing different aspects of patient care[53].

### Improvement

Patient-rated improvement is an important, patient-oriented outcome measure that has been demonstrated to be reliable and responsive[77-79]. Patients are asked to use the 9-point scale to compare their BRLP condition to what it was before study treatment. Response choices are No symptoms (100% improvement), Much better (75% improvement), Somewhat better (50% improvement), A little better (25% improvement), No change (0% improvement), A little worse (25% worse), Somewhat worse (50% worse), Much worse (75% worse), and Twice as bad (100% worse).

### Medication Use

Non-prescription and prescription medication use are measured using a 5-point scale. Subjects indicate how frequently they have taken medication for their BRLP in the past week. These measures have been used in two previous studies by Bronfort et al[36,53]. To assess the accuracy of medication documentation, patients are asked to bring in their medications at the first baseline evaluation and the week 12 evaluation.

### EuroQol 5D

The EuroQol is a multi-attribute utility scale covering five dimensions (mobility, self-care, usual activities, pain/discomfort, and anxiety/depression) with three levels (no problem, moderate problem, severe problem)[80,81].

### Self-efficacy

Self-efficacy is measured by the Pain Self-Efficacy Questionnaire, a 10-item scale used to assess the level of self-confidence in performing functional and social activities despite the presence of pain. Each item is rated from 0 (Not at all confident) to 6 (Completely confident) and scores range between 0 (no self-efficacy) and 60 (highest self-efficacy)[63,82].

#### Biomechanical and Clinical Objective Measures

A set of six different tests are performed in a single session at BEV2 and repeated at week 12. All objective testing is performed by examiners trained and certified in testing protocols and blinded to patients' treatment assignment. The tests consist of the following:

### Continuous Lumbar Motion

Lumbar motion is assessed using an electromagnetic tracking system (Polhemus Liberty, Colchester, Vermont) and motion monitor software (Innovative Sports Training Inc, Chicago, Illinois), which yields accurate measurement[83]. We have found the angular measurements to be accurate within one degree in our laboratories when compared with a mechanical angular protractor device. Electromagnetic sensors are attached at the thorax and the sacrum using hard plastic plates held in place over bony landmarks with straps. The system acquires three-dimensional position and orientation from the electromagnetic sensors and the relative motion is computed between the trunk and the sacrum using Euler angles. Data are collected using a modified protocol described by Vogt et al[84] for flexion-extension, rotation, side-bending, and circumduction at a sampling rate of 120 points per second. Data reduction is performed using MathCAD software (Mathsoft Inc., Waltham, MA, USA) at PCCR to obtain the following parameters: (1) maximum ranges of motion in the sagittal, coronal, and axial planes and (2) average velocities in the sagittal, coronal, and axial planes from neutral to end ranges.

### Standing Postural Sway

Postural sway data are collected using a method and protocol developed by Bhattacharya et al[85] and adapted for use at PCCR. Patients are blindfolded and stand on a force plate (Model # 4060-NC, Bertec, Inc, Columbus OH) for 30 seconds with a safety harness secured to their torso and attached loosely to the ceiling. Three forces and three moments are collected from the force plate at a rate of 1000 points per second using Motion Monitor software (Innovative Sports Training, Inc, Chicago, IL), which also calculates the x (fore-aft) and y (side-to-side) coordinates of the participant's center of pressure (COP) location, based on dividing the flexion-extension moment by the vertical force and dividing the lateral-bend moment by the vertical force, respectively. The test is repeated with a 10 cm thick section of latex foam placed on top of the force plate. This cycle of measurements is repeated twice, providing four, 30-second COP recordings.

The PCCR Biomechanics Team performs data reduction for patients tested at both sites. For each 30-second segment of data collection, 30,000 points of x-y coordinate data are reduced into 4 COP variables: (1) maximum radial distance the COP traveled from the average location, (2) mean radial distance from the average, (3) total area covered by the COP as it moved during those 30 seconds, and (4) total distance the COP traversed in mm during the 30 seconds. Hence, this assessment produces 8 values--4 from each of 2 different 30-second segments of data collection. With 2 measurement cycles of these data, a total of 16 values are determined from each participant. Reduced data are transferred to the DCC for analysis.

### Neuromuscular Response to a Sudden Unexpected Load

Data are collected using methods and protocols developed by Wilder et al[86] and adapted for use at PCCR that measure muscle activity and COP changes associated with the immediate response to a sudden unexpected force pulling from the participant's chest. EMG electrodes are attached over the paraspinal muscles of the participant bilaterally at the L3 level and connected to an EMG amplifier (Delsys, Inc. Scottsdale AZ). While standing on a forceplate (Model # 4060-NC Forceplate, Bertec, Inc, Columbus OH), participants are fitted with a harness strap around their back at the midsternal level. A load cell (Omegadyne LC101-100, Omegadyne, Inc., Sunbury OH) and accelerometer (Triaxial CXL10LP3, Crossbow Technology, Inc. San Jose, CA) are rigidly attached to the harness. A cord attaches the load cell via a pulley to a 1.8 kg weight that hangs in a PVC tube. The pulley and tube are rigidly attached to a firm concrete wall. The height of the pulley is adjusted so that the cord is horizontal. The participant is blindfolded and wears earphones with loud radio carrier noise to prevent cueing. The weight is raised in the tube between 25 and 35 cm and suspended by a second rope held by the operator's hand. At a signal from the computer operator, the weight is dropped to introduce a sudden tug to the patient's upper trunk.

The Motion Monitor software is triggered by the load cell to record a 4-second segment of data from the two EMG channels, the load cell, accelerometer, and force plate at a rate of 1000 samples/sec. A trial drop is performed with the patient's eyes and ears uncovered to ensure they can tolerate the impact and that an adequate EMG signal is recorded. The force of the tug to the chest depends on the height from which the weight is dropped. The drop distance is adjusted for each patient based on their weight and to ensure that a clear activation of the back muscles can be seen on EMG. Six repetitions of the drop are performed with the blindfold and earphones masking any cues to provide an "unexpected" sudden load.

Raw data collected from both sites is reduced at PCCR using custom software written by Lee[87].

Sixteen variables are collected from each drop including 6 variables that describe the imposed load and subsequent participant movement and 5 paired (left and right sided) paraspinal EMG response factors. The 6 loading and movement factors include: 1) peak force exerted on the participant's trunk, 2) time to that peak force 3) the peak acceleration of the participant's trunk, 4) the time to that peak acceleration, 5) excursion of the participant's COP in the fore-aft direction, and 6) the time to that maximum COP excursion. The EMG factors are: 1) amplitude before the sudden load, 2) length of time from the tug on the harness to the beginning of the EMG responses, 3) magnitude of the maximum EMG responses, 4) time to maximum EMG responses, and 5) duration of the EMG responses.

### Lumbar Paraspinal Muscle Flexion-Relaxation

Our methodology, based on the work of Watson et al,[88] combines the EMG electrode setup of the sudden load test above and the electromagnetic tracking of the continuous lumbar motion to measure the spinal motion and activity of the lumbar paraspinal muscles during a forward bending task. Participants are instructed to move from an upright standing posture into full forward flexion in a smooth manner over 6 seconds. Full flexion is maintained for 1 second followed by a return to the upright position over another 6 seconds. After a 3-second rest, the movement is repeated. A total of three cycles of EMG and position are recorded.

EMG and position data are processed using MathCAD software and custom routines. The EMG signal is rectified and the RMS calculated with a 100 ms window to produce continuous traces of left and right activity with respect to time. The position channels are reduced to provide the lumbar flexion angle. Semi-automated routines locate the maximum EMG activity during the forward flexion task and the minimum activity during the period when the trunk is fully flexed. The flexion/relaxation ratio is calculated as the minimum flexed EMG divided by the maximum during flexion. Previous research has shown that the lumbar paraspinal muscles normally become electrically silent at full forward flexion, whereas patients with low back pain often fail to show this silent period. The flexion/relaxation ratio is a factor that will be used to show the extent to which patients with BRLP exhibit the silent period and whether this factor changes with treatment.

### Torso Muscle Endurance

Blinded examiners measure muscle endurance of the trunk flexors, lateral flexors, and extensors. Tests are performed following a protocol described by McGill[89]. These tests have been shown to be valid and reliable measures of torso muscle endurance[90,91]. Test data consist of the time that each posture is held (in seconds), which are used to calculate ratios. The measurements provide an objective measure of treatment outcome and a baseline guide to the individualized home exercise program.

The EMG sensors are left in place for the extension task of the endurance test. This provides a measure of EMG activity with a known load (trunk weight) to enable calibration of the EMG signal to help with interpretation of the sudden load test; it also provides a measure of EMG activity during fatigue.

### Straight Leg Raise Test

To assess the presence of nerve root irritation, the straight leg raise test is performed by blinded examiners using a digital inclinometer to record angle of leg elevation producing leg symptoms. The straight leg raise test has acceptable reliability[92] and some evidence of validity[93]. With the patient in the supine position, the inclinometer is placed just proximal to the patella and zeroed with the leg in the neutral position. The lower leg is then extended passively until the knee is in full extension; then the whole leg is raised off the table until the patient indicates pain[92]. Test data consists of angle of leg elevation at pain onset and the site of pain.

#### Other Measures

##### Patient Expectation of Care

Prior to treatment group assignment, patients are asked to rate how helpful they think each treatment group will be for their BRLP using an 11-box scale (0 = not at all helpful, 10 = extremely helpful)[94]. Prior to treatment, patients are asked to rate their expectation of improvement using the improvement scale described above.

##### Side-effects

Patients are asked to report side-effects in the patient self-report questionnaires by responding to a list of side-effects generated from previous studies by the investigators assessing SMT and self-care education[42,49]. For each side-effect listed, the patient is asked to indicate if they experienced it, and if yes, to rate the bothersomeness of the side-effect on an 11-box scale (0 = not at all bothersome, 10 = extremely bothersome). This method of recording side-effects is an attempt to standardize side-effect reporting in clinical trials, which has been inadequately addressed in much of the research performed to date[31].

##### Potential Confounding Variables

Health care utilization (dates and types of services for BRLP, type and dose of rescue medications), compliance with the study interventions, and patient expectations is documented in the patient self report questionnaires and the patient file. Clinical depression may also be a confounding variable and is assessed by the Community Epidemiologic Scale-Depression (CES-D), designed for non-psychiatric patients[95]. This one page questionnaire, consisting of 20 depression related questions, was developed by the National Institutes of Health and has been found to be reliable and valid[96].

##### Patient Interviews

Face-to-face interviews are conducted on an individual basis, after the 12 week treatment phase. A schedule of questions is used to direct the interviews and keep the interviewers on a path consistent with the purpose of the study[97]. The questions begin broadly, asking how patients felt about the treatment they received, whether it met their expectations, and what they liked and disliked. These questions are then followed by probe questions to elicit underlying reasons. The format of the interviews is semi-structured with open-ended questions. Permission is sought to tape record the interviews, and participants are assured confidentiality, allowing them to speak freely in response to the questions[98]. All interviews are transcribed for analyses.

To ensure consistency with interview techniques, staff from both sites are trained using standardized protocols[97]. Random samples of 10% of interviews from each site are compared to recorded interviews for accuracy.

##### Data Management

The Data Coordinating Center at PCCR's Office of Data Management and Biostatistics handle all study data. The DCC database system uses password-protected web-based transfer protocols to collect patient management information across sites.

### Statistical Methods

The DCC biostatisticians will conduct data analyses using SAS^® ^(Release 9.0) and S-Plus 7.0 for Windows. Descriptive statistics of patient characteristics will be presented for each treatment group to assess their comparability as well as the generalizability of the sample.

### Data Analysis

Effects in patient-rated leg pain between groups will be assessed by modeling over weeks 3, 12, 26, and 52 adjusting for the baseline value of leg pain and the baseline variables used in the minimization algorithm for treatment allocation. Mixed models, using SAS Proc Mixed, will be used to examine patterns and estimate effects between groups after fitting models that account for the correlation across measurements in the same patient and using the variance-covariance structure that best fits the data[99,100]. Normality assumptions will be evaluated through normal probability plots and transformations explored if necessary. We will test for site-by-group and time-by-group interactions. A level of significance of 0.05 will be used to judge the results as statistically significant. Adjusted mean differences and 95% confidence intervals between groups at weeks 3, 12, 26, and 52 will be presented in tables and line graphs. Further adjustment will control for unbalanced baseline and other possible confounding variables to increase precision of the estimates. An intention-to-treat analysis will be used; all patients with at least one follow-up measure will be included in the analysis, as the methods do not require data at every time point.

### Sample Size

Sample size projections were based on a power analysis using SAS Proc GLMPower. In the latest pilot study by WHCCS, group differences in pain of 8 percentage points after 3 months of treatment were observed[42]. Informed by these results, the scientific literature,[31,101] and consensus of study investigators and clinicians, we are interested in detecting an 8 percentage point difference between the two group means in patient-rated leg pain at the short- and long-term. In our pilot study, correlation coefficients among the pain variable over time varied from r = 0.22-0.25 and estimates of standard deviation were 1.7 (scale 0-10).

Using these estimates, the sample size calculation based on the analysis of covariance at one time point, adjusting for the baseline value, indicates 80 patients per group are required to achieve 86% power at the 0.05 level of significance to detect a difference between groups of 8 percentage points.

The same data analysis approach will be used to analyze the secondary outcome variables as confirmatory analyses to assist with the interpretation of study results. A more conservative Bonferroni-adjusted level of significance will be used to judge the results as statistically significant.

### Missing Data

Every effort is made to prevent the occurrence of missing data. The mixed model analysis includes all patients that have at least one follow-up assessment. To examine the possible effects of missing data on the results, SAS Proc MI will be used to produce multiple imputation analyses. The Markov Chain Monte Carlo approach will used to impute all missing values of the outcome variables from baseline demographic characteristics and the baseline primary and secondary outcome measures. We will draw 5-15 imputations and fit the same mixed model analysis described above. SAS Proc MIAnalyze will be used to combine the results to obtain estimates of regression coefficients, standard errors and p-values[102,103].

### Analysis of Biomechanical Measures

First, baseline measures of continuous spinal motion, postural sway, response to sudden load, and flexion-relaxation in the patients with BRLP will be compared to data (unpublished) from previous trials of patients with sub-acute and chronic low back pain *without *radicular pain. Next, we will explore effects of these measures after 12 weeks of active care, taking into account gender and body mass index. If there are unbalanced baseline or other confounding variables, we will also control for these in the analysis. Finally, we will examine and describe the bivariable relationships between our primary outcome, leg pain, and each biomechanical variable.

### Patient Interview Analyses

Content analysis using an inductive approach[104] will be used to identify categories and themes that occur in the transcribed text generated from the qualitative interviews[105].

The text of ten interviews will be read independently by two investigators from each site to gain an overall impression and to establish and define preliminary codes in response to the proposed questions[106,107]. After the initial analysis, the investigators will meet to reach a consensus on preliminary codes. These codes will be entered into a "code book," which will provide a detailed definition for each code[105]. Information related to methodological decisions and their rationale will also be documented[104]. Subsequent transcripts will be independently reviewed to identify and code text segments and to assess the inter-rater reliability of text coding. Kappa values of less than 0.8 will necessitate review of the coding structure.

Categorized information from the transcribed interviews will be entered in matrices to organize and display categories by treatment groups as a means of illustrating relationships among categories[108]. This information will then be summarized and interpreted.

## Discussion

Back-related leg pain (BRLP) is a costly and often disabling variation of the ubiquitous back pain conditions. As health care costs continue to climb, the search for effective treatments with few side-effects is critical. While SMT is the most commonly sought CAM treatment for LBP sufferers, there is only a small, albeit promising, body of research to support its use for patients with BRLP.

This study is unique in that it is one of the first full scale randomized clinical trials to evaluate SMT for the potentially costly and disabling BRLP conditions. By using a two site design, this study capitalizes on the complementary strengths of each participating institution, enhances generalizability of study results, and facilitates recruitment efforts.

Importantly, our study is designed to provide results that are relevant and meaningful from both research and clinical perspectives. We have chosen to combine SMT with patient education compared to patient education alone for the following reasons: first, it is pragmatic, because chiropractic care delivered in general practice usually includes SMT with some type of education[28]. Second, standardizing patient education enhances the investigators' ability to measure SMT's unique contribution to treatment outcome. Finally, by having the same patient education administered in both groups, we can control for the differential non-specific effects that may be associated with patient education. Also, there are several advantages to using patient education as a minimal intervention comparison. Patients expect to receive information and advice about their LBP condition[109]. As an intervention, it is credible to patients (in comparison to no treatment, waiting list, or sham treatment), and it can enhance patient compliance in clinical trials[41,42,49].

This study seeks to fill a critical gap in the LBP literature by performing the first full scale RCT assessing chiropractic SMT for patients with sub-acute or chronic BRLP using important **patient-oriented **and **objective biomechanical **outcome measures. Upon completion, this study will provide clinically useful information regarding chiropractic care and home exercise for the understudied sub-population of LBP--those with BRLP.

## Competing interests

The authors declare that they have no competing interests.

## Authors' contributions

The authors are investigators of the study and each contributed to the study design. GB, MH, and RE are primarily responsible for the conceptualization of the trial. GB is the principal investigator of the HRSA grant award, and MH is the site principal investigator. CS prepared the first draft of the manuscript and organized revisions. MG, EO, DW and CL provided information for the methods section. All authors read and approved the final manuscript.
